# Effects of diabetes mellitus on step length and minimum toe clearance adaptation

**DOI:** 10.1186/s12938-023-01082-2

**Published:** 2023-05-10

**Authors:** Suzanne Martin, Simon B. Taylor, Blynn L. Shideler, Rajna Ogrin, Rezaul Begg

**Affiliations:** 1 Institute for Health and Sport, Victoria University, Melbourne, VIC 3011 Australia; 2 School of Medicine, Stanford University, Stanford, CA USA; 3 Bolton Clarke Research Institute, Melbourne, VIC Australia

## Abstract

**Background:**

Adaptive gait involves the ability to adjust the leading foot in response to the requirement of dynamic environments during walking. Accurate adjustments of the minimum toe clearance (MTC) height and step length can prevent older people from falling when walking and responding to hazards. Although older people with diabetes fall more frequently than healthy older adults, no previous studies have quantified their adaptive gait abilities. This study aimed to investigate the effects of diabetes mellitus on step length and MTC height adjustments using a non-immersive virtual-reality system.

**Methods:**

Sixteen young adults (26 ± 5 years, 7 females), 16 healthy older adults (68 ± 5 years, 6 females), and 16 older adults with diabetes (70 ± 5 years, 6 females) completed adaptability tests while walking on a treadmill. A computer system visualised a continuous real-time signal of absolute step length and MTC on a monitor. Each person responded to four discrete participant-specific step length and MTC visual targets that were presented on the same signal. Tasks were to match the peaks of interest on each signal to presented targets. Targets were 10% longer or shorter than the mean baseline step length, and 2.5 cm, and 3.5 cm higher than the mean baseline MTC. When a target was displayed, it remained unchanged for 10 consecutive foot displacement adaptation attempts. Then, the target was removed and a new target or the same target was present after 10 consecutive steps and remained for 10 steps. Each target was randomly presented three times (3 × 10). Step length and MTC height adjustments in response to targets were measured and compared among groups.

**Results:**

Mean preferred walking speeds were not different among groups significantly when no targets were presented on the monitor in baseline walking. However, when participants walked on a treadmill while attempting to match step lengths or MTC heights to displayed targets on the monitor, the group with diabetes had reduced step length and MTC adjustments compared with other groups significantly. They showed greater errors (differences between their step lengths/MTC heights and presented targets) on the monitor.

**Conclusions:**

This study quantified reduced abilities for older individuals with diabetes to adjust both step length and MTC in response to stimuli compared to healthy older counterparts. Reduced step length and MTC height adjustments can increase falls in at risk populations. The presented virtual-reality system has merits for assessing and training step and MTC adaptation.

## Background

Falls constitute one of the major causes of morbidity and mortality in older adults, and in older people living with diabetes, falls tend to occur more frequently leading to more fall-related injuries and increased likelihood of early mortality. International figures indicate that approximately 28–35% of adults aged 65 and over fall each year, increasing to 32–42% in adults 70 years and older [1]. Diabetes is an independent risk factor for falls in older adults, with a 64% greater risk of falls, compared to older adults without diabetes [2]. Falls in adults over the age of 65 are the leading cause of unintentional injury and account for 40% of all injury-related deaths [3]. Eighty-seven percent of older adults with diabetes experience a fall [4], in which 30–50% of these falls cause minor injuries, and 5–10% cause major injuries, such as the fracture of the neck of the femur [5]. A quarter of older people with a femur fracture die in a year, half reduce their daily activities, and 22% move into long-term care [6].

The economic consequences of falls are significant, with direct annual medical cost of $23.3 billion in the United States and US$1.6 billion in the United Kingdom [7]. Fall-related medical events account for 40% of long-term care placements and contribute to a further increase in healthcare costs [8]. In 2000, the Australian Institute of Health and Welfare predicted that the cost of fall-related hospitalisation would increase, requiring an estimated 3,320 long-term care places in 2051 [9]. Given the significant impact on mortality, morbidity, and economics, investigating the factors can reduce fall-related injuries in older people with diabetes is warranted.

One of the main reasons for falls in at risk populations is the inaccuracy of foot adjustments in response to the sudden appearance of obstacles and stepping targets [6, 9–11]. A fall mostly occurs during walking when responding to a perturbation (e.g., obstacle), highlighting the decreased ability to precisely respond to external hazards. Tripping over an obstacle is responsible for 41% of fall incidents in older people [9] when individuals are unable to adjust their feet before, during and after obstacle negotiation. Therefore, their foot may contact the obstacle, and trigger a fall [6, 10, 11].

People who are older in age show reduced accuracy of foot adjustments in response to obstacles and stepping targets [10, 12–16]. Older people were unable to accurately place their feet in response to goal-oriented tasks compared with young adults in previous research [10, 12]. While negotiating obstacles, the older adults contacted obstacles or used compensatory strategies to avoid the obstacles [10, 14]. Research is equivocal, with some investigators reporting groups with older adults touching obstacles more than the young group [11, 17–19], with other investigators reporting older adults avoiding obstacles as well as young people [20–22].

The existing knowledge about foot adjustments in older adults with diabetes has been built on a few studies that reported a decrease in toe-obstacle clearances, where participants did not touch any obstacles [23–25]. This may imply that the task of obstacle avoidance might not be sufficiently challenging; the obstacle was always visible before participants started walking towards it. Since a static obstacle obstructed the walkway, participants could adapt their step lengths a few steps ahead to place the obstacle in the middle of their crossing steps without any risk of touching it.

Assessing and training foot adjustments using visual cues (ground-projected targets) and non-immersive virtual-reality targets have shown a successful method for reducing falls in Stroke, Parkinson’s disease, and older fallers [13, 26–29]. However, there is limited knowledge of their use in older people with diabetes. Computer systems can quantify and train more accurate foot adjustments in biomechanics laboratories for safe ambulation of risk populations during daily life activities. This paper provides evidence for the use of non-immersive virtual-reality targets [26] to measure the accuracy of foot displacement adjustments in the sagittal plane. Some tools have been developed to visualise meaningful Vicon data collected from shoes’ tracking retroreflective markers in real time and virtual targets on a monitor [30].

The aim of this study was to investigate whether the abilities to adjust step lengths and minimum toe clearance heights when seeing stepping targets/obstacles would be reduced in people with diabetes. This study also presented potential merits of the new instrument [26] that quantifies the accuracy of responses to virtual stepping targets/obstacles during treadmill walking as assessment tools to refer people at an increased risk of falling for interventions.. It was hypothesised that the accuracy of step length and MTC adaptation would be the same between older adults without diabetes and older adults with diabetes who had not developed diabetes-related neuropathy.

## Methods

For separating the effects of age and diabetes mellitus on the accuracy of foot displacement adjustments in response to the sudden appearance of virtual targets, a priori power calculation calculated that 16 young adults (Group I), 16 healthy older adults (Group II), and 16 older adults with diabetes (Group III) would require to detect an effect size of 0.66 with 95% power and a significant level of 0.05. Therefore, the study included 48 participants, older and young adults aged 60–85, and 20–35, respectively.

All groups were active and had no health issues except group II lived with diabetes for more than 5 years and took an oral medication (Metformin). Participants had no self-reported fall in the year before participating in the study. The following tests were also completed for each participant before being included in the study: (i) visual acuity: the vision of each participant was examined using an eye chart; (ii) cognitive function: the mini-mental state examination (MMSE); (iii) neuropathy: the Michigan neuropathy screening instrument (MNSI) which was used comprises two sections: questionnaire and clinical examination; (iv) kicking a soccer ball from a standing position [44]: the leg that was used to kick the ball was called the dominant leg; and (v) observational gait assessment. Participant exclusion criteria were: vision acuity less than 20/40; cognition scores less than 27; neuropathy scores of 3 and over; and any gait deviations.

**Fig. 1:**
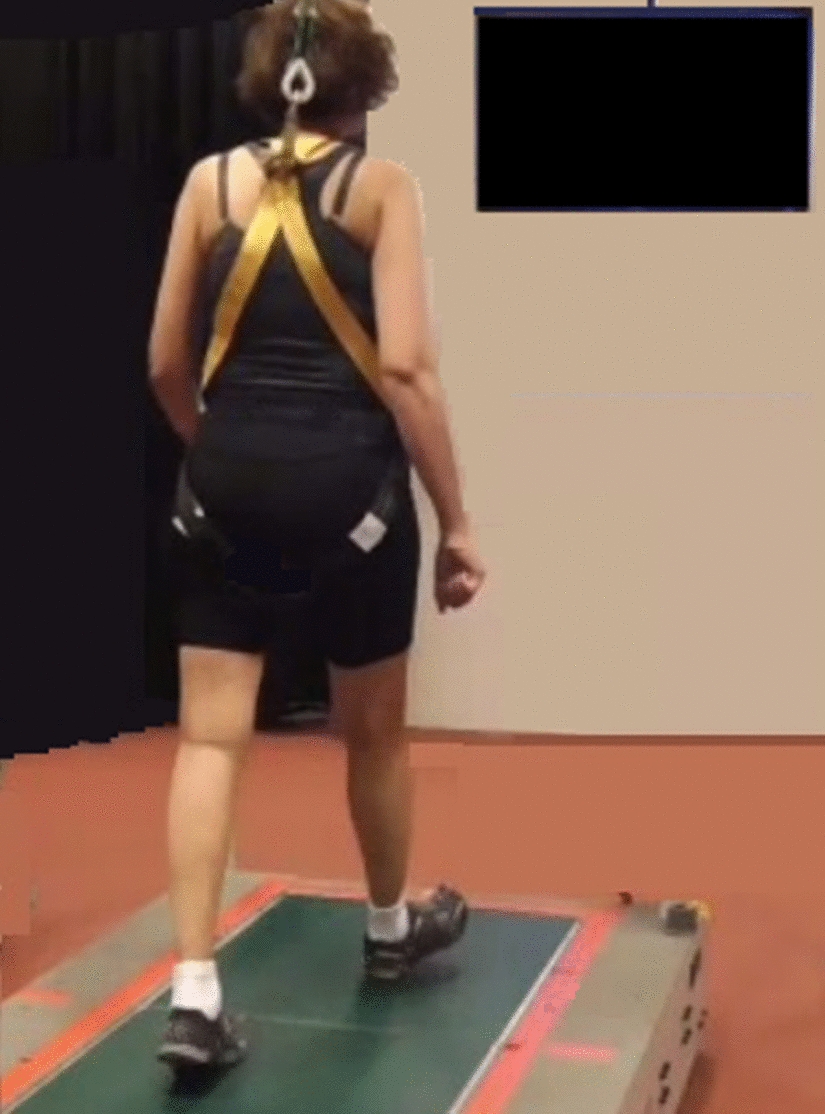
Baseline tests. The participant walked at her determined preferred speed without seeing anything on the monitor (black screen)

The study was conducted with the permission of a human ethics committee (HRE17‐194). A Vicon motion capture system collected kinematic data of two retroreflective markers attached to provided sports shoes (Merrell Bare Access 4) on the distal areas of the first toes. The system streamed the data in real-time into a customised MATLAB program (Mathworks, Natick, Massachusetts, USA) via Visual3D-Server software (C-Motion, Germantown, Maryland, USA) [26, 30]. Participants wore the provided sports shoes and a safety harness connected to a frame placed around a treadmill (Fig. 1).

**Fig. 2:**
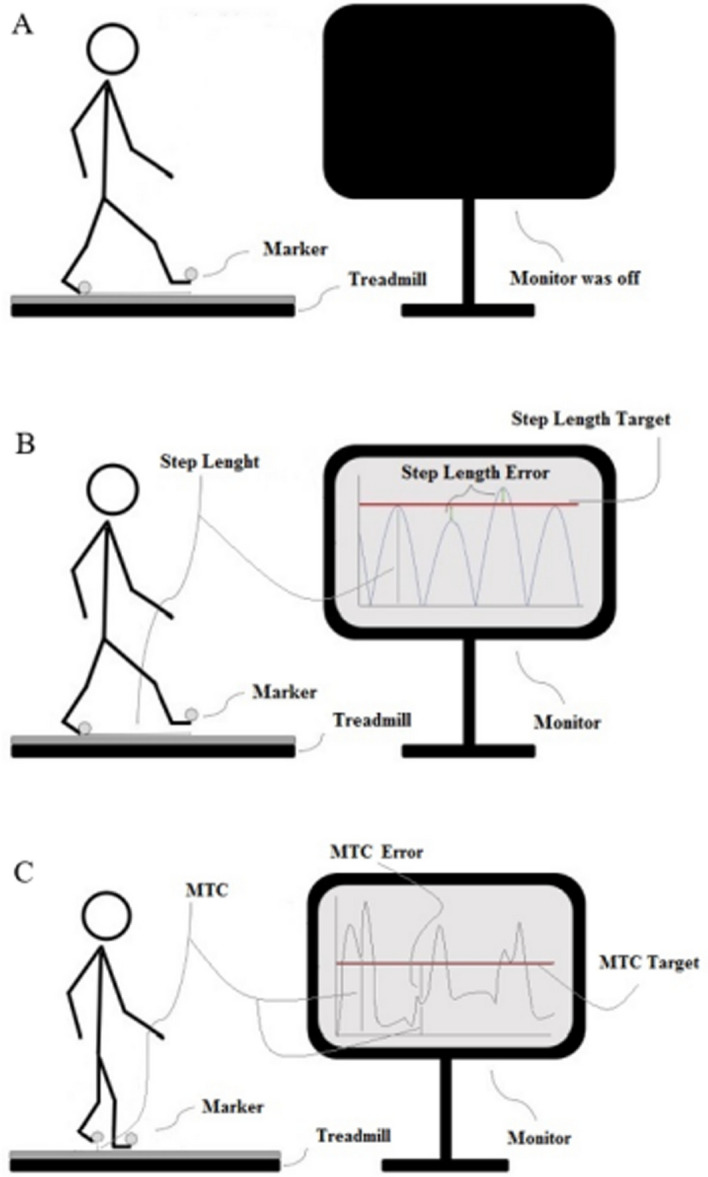
Baseline and adaptability tests. In Baseline (**A**), the monitor was switched off when participants walked at their preferred speeds for 10 min. In step length adaptation (**B**), the monitor displayed a line (step length target) and participants were tasked to match peaks of step length line graphs with the line on the screen, until the line was removed. In minimum toe clearance (MTC) height adaptation (**C**), the monitor displayed a line (MTC target) for 10 steps, and the task was to match the lowest peak before the highest peak by lifting the foot higher

Participants walked with several speeds (0.8–1.8 m/s) for 3–5 min before determining their preferred speed. For each person, the preferred walking speed was determined as the average of the three highest and lowest uncomfortable walking speeds [31]. Participants then walked without any instruction for 10 min in a baseline condition when the monitor installed in front of them at their eyes’ levels was switched off (Fig. 2A). During this time, a MATLAB script was run three times to use three 60-s gait data collected by VICON for calculating the mean step length and MTC height of each foot.

The MATLAB script computed mean step length and MTC that determined four virtual targets in the baseline. Stepping targets were 10% longer and shorter than the mean step length; MTC targets were 2.5 cm and 3.5 cm higher than the mean MTC. The method of determining targets was tested in a group of young adults who achieved minimum errors in response to targets [26].

In the adaptation tests, real-time step length or MTC displayed as a line graph on the monitor (Fig. 2B, C). The real-time step length was the absolute difference between the two toe marker positions in each step (the peak of the step length graph), and the real-time MTC height was the lowest peak of the toe marker of the swing leg before reaching its maximum height in the vertical direction. A target (the red line in Fig. 2) appeared on the monitor for 10 consecutive steps. The target was then withdrawn for the following 10 steps. After two–four steps without seeing any target on the monitor, participants walked with their average step length/MTC [13]. The same or a new target was presented for the next 10 steps and removed for the following 10 steps. Each target appeared three times and disappeared three times in random order. Participants used real-time visual information to minimize the distance between their executed step length or MTC and a presented target (Fig. 2) by matching the points of interest with presented target lines. Participants were unaware of targets’ values and orders; however, they knew the number of trials and durations of targets’ appearance and disappearance.

The error of each response was determined (number of responses = 120). Constant and absolute error in each step was calculated as follows:

$${{\text{Step length}} \mathord{\left/ {\vphantom {{\text{Step length}} {\text{minimum toe clearance}}}} \right. \kern-0pt} {\text{minimum toe clearance}}}{-}{{\text{Step length}} \mathord{\left/ {\vphantom {{\text{Step length}} {\text{inimum toe clearance target}}}} \right. \kern-0pt} {\text{inimum toe clearance target}}} = \, {{{\text{Short}}} \mathord{\left/ {\vphantom {{{\text{Short}}} {\text{long constant error}}}} \right. \kern-0pt} {\text{long constant error}}}$$

$$\left| {{{\text{Step length}} \mathord{\left/ {\vphantom {{\text{Step length}} {\text{minimum toe clearance}}}} \right. \kern-0pt} {\text{minimum toe clearance}}} \, {-}{{\text{Step length}} \mathord{\left/ {\vphantom {{\text{Step length}} {\text{minimum toe clearance target}}}} \right. \kern-0pt} {\text{minimum toe clearance target}}}} \right| = \, {{{\text{Short}}} \mathord{\left/ {\vphantom {{{\text{Short}}} {\text{long absolute error}}}} \right. \kern-0pt} {\text{long absolute error}}}$$

The first response of each block of 10 steps (reactive responses) was removed [31]. A statistical analysis software was used (Version 25 for Windows, SPSS Science, Chicago, Illinois, USA) at a significance level of *α* = 0.05. Nonparametric tests (Kruskal–Wallis *H* and Mann–Whitney *U*) were used to compare absolute and constant step length and MTC errors among groups.

## Results

Older participants with diabetes had a mean glycated haemoglobin A1c (GHbA1c) of 7.6% ± 1.8%. None of the participants experienced a fall in the year before their participation, and all of them achieved a score of 27 and over when the MMSE was completed for them. Two older adults with diabetes were excluded, because they had peripheral neuropathy (MNSI score ≥ 3). All participants kicked the soccer ball with their right legs. Two healthy older participants and one older participant with diabetes were excluded, because they were uncomfortable walking on the treadmill. In total, 16 young adults, 14 healthy older adults, and 13 older adults with diabetes completed baseline and adaptability tests during treadmill walking.

Table 1 presents the characteristics of participants in each group. Preferred walking velocities were 1.08 m/s (SD = 0.12 m/s) in young adults, 1.08 m/s (SD = 0.16 m/s) in healthy older adults, and 0.98 m/s (SD = 0.11 m/s) in older adults with diabetes. They were not different among groups significantly.

**Table 1:** Participants ‘characteristics include mean ± standard deviation of age, body mass, and height in young adults (Group I), healthy older adults (Group II), and older adults with diabetes (Group III)

	Group I (*n* = 16)	Group II (*n* = 14)	Group III (*n* = 13)
Participants	9 males, 7 females	8 males, 6 females	7 males, 6 females
Age (years)	26.06 ± 4.97	68.36 ± 5.43	69.62 ± 4.81
Body mass (kg)	75.61 ± 9.05	75.04 ± 9.75	76.67 ± 11.14
Height (cm)	175.00 ± 6.40	167.93 ± 10.84	167.23 ± 9.97

**Fig. 3:**
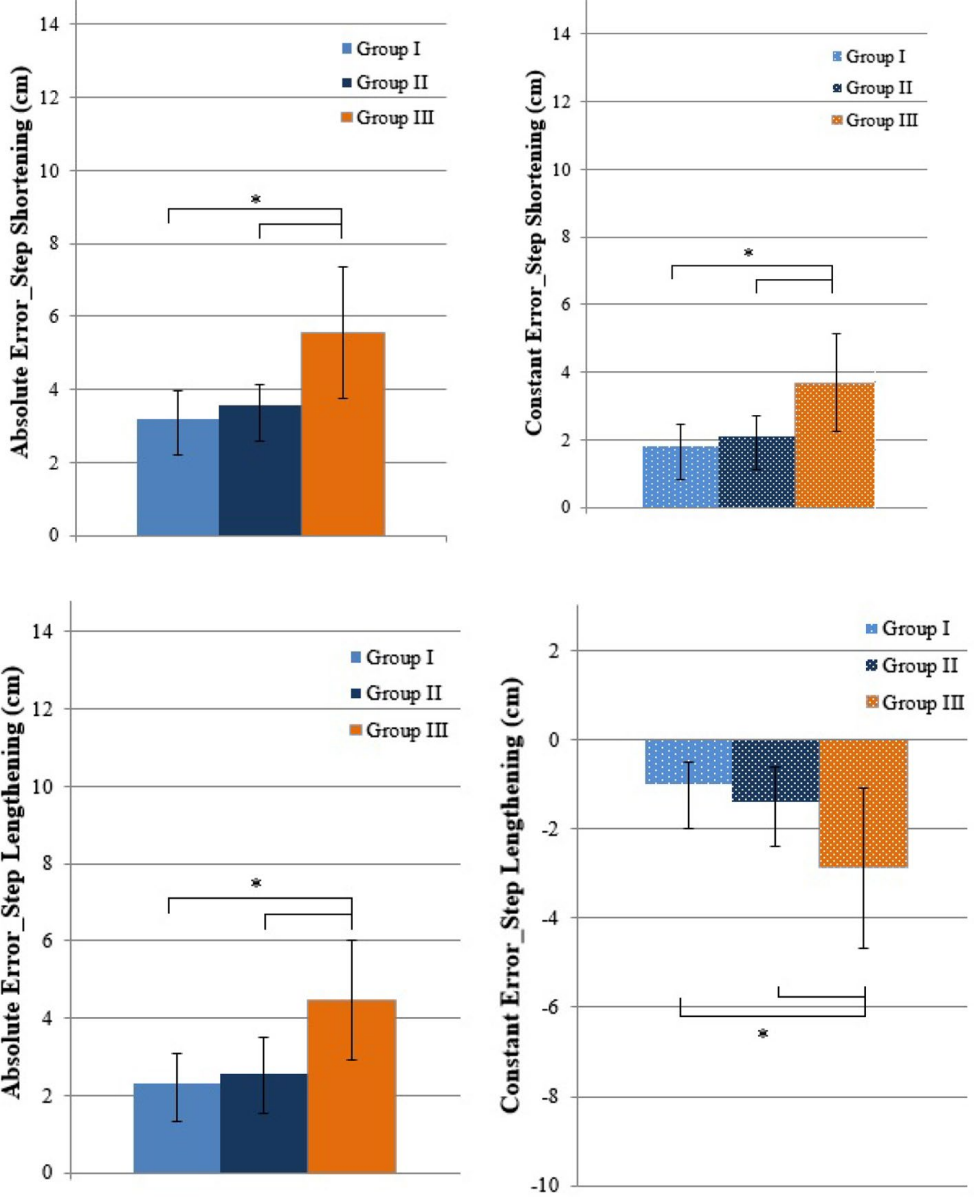

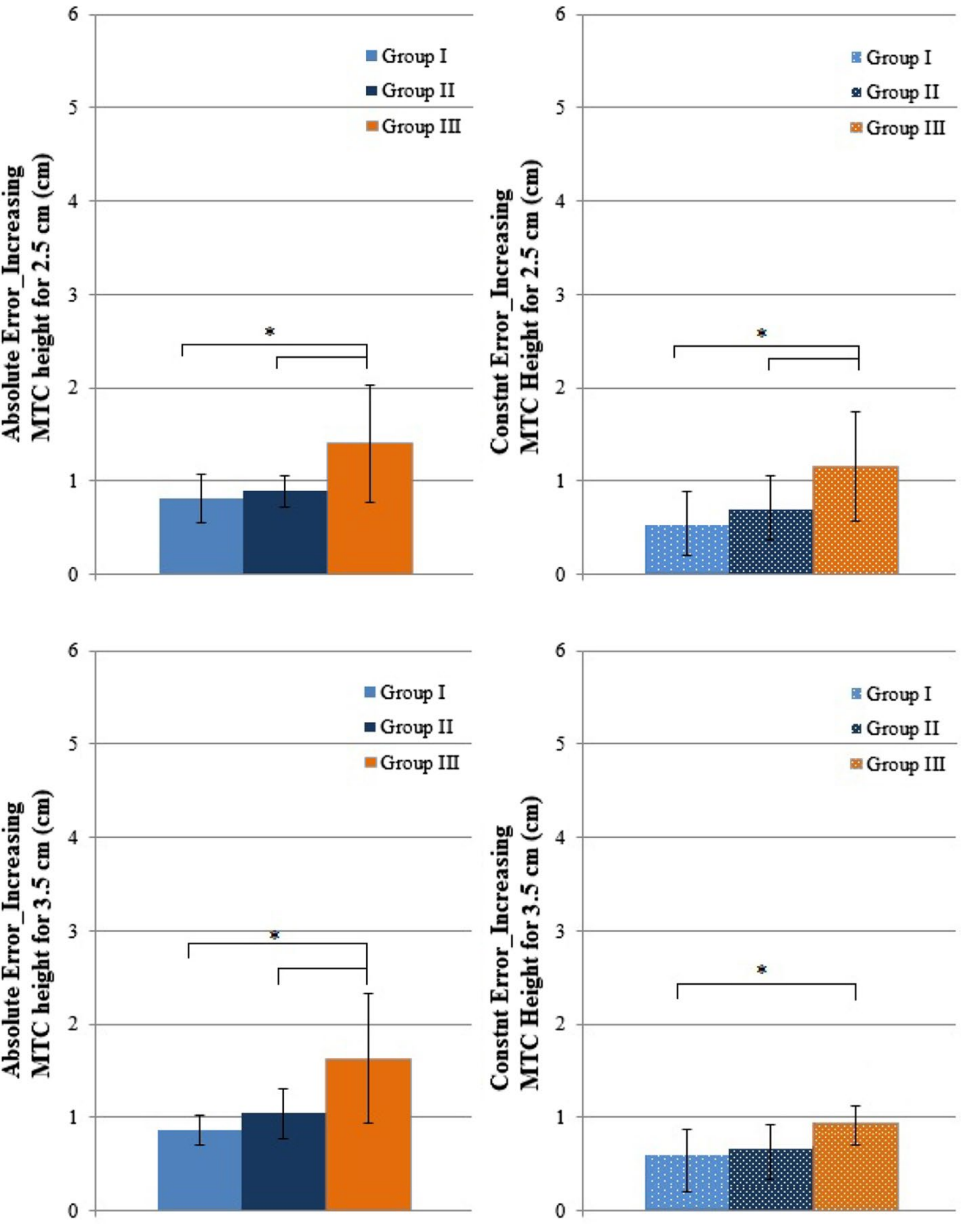
Mean and standard deviation of constant and absolute errors in response to four targets for step shortening, step lengthening, and increasing minimum toe clearance (MTC) high for 2.5 cm and 3.5 cm in Group I (young), Group II (older), and Group III (older diabetes). Significant differences between groups (*)

Figure 3 presents constant and absolute errors during step shorting, step lengthening, and increasing the MTC height in response to presented targets on the monitor in each group.

### Step shortening

As Fig. 3 shows, groups’ mean absolute errors during step shortening were significantly different (*p* = 0.0004). Mean absolute errors of step adaptation during step shortening of Group I and Group III were different (*p* = 0.0003) and Group II (2.8 cm) and Group III (5.8 cm) (*p* = 0.004). Mean constant errors were different among groups (*p* = 0.002). Mean constant errors in Group III (3.8 cm) were significantly higher than those in Group I (*p* = 0.0002) and Group II (*p* = 0.004). *Step lengthening.*

Groups’ mean absolute errors during step lengthening were different (*p* = 0.001) (Fig. 3). Mean errors of Group I (2.2 cm) and Group III (4.3 cm) were significantly different (*p* = 0.001) as well as Group II (2.3 cm) and Group III (*p* = 0.008). Mean constant errors were negative and different among groups (*p* = 0.003). Mean constant error in Group III was larger in magnitude than in Group I (*p* = 0.001) and Group II (*p* = 0.002). *MTC height increasing for 2.5 cm.*

Mean absolute and constant errors when adapting the MTC with the presented targets were different between the groups (*p* = 0.0004 and *p* = 0.004) (Fig. 3). Mean absolute errors of Group I (0.8 cm) and Group III (1.4 cm) were different (*p* = 0.0003) and Group II (0.9 cm) and Group III (*p* = 0.004). Mean constant errors in Group III were significantly higher than those in Group I (*p* = 0.004) and Group II (*p* = 0.004). *MTC height increasing for 3.5 cm.*

MTC Mean absolute and constant errors during walking with increased MTC heights were significantly different among groups (*p* = 0.004, *p* = 0.004) (Fig. 3). Group III had higher absolute errors than Group I (1.6 cm) and II (1 cm) (*p* = 0.002 and *p* = 0.007). The mean constant error in Group III (0.9 cm) differed from that in Group I (0.5 cm) (*p* = 0.004) and was insignificantly different from the mean constant error in Group II (0.9 cm).

## Discussion

We investigated the effects of diabetes mellitus on step length and minimum toe clearance adaptation using a computer system that presented continuous real-time step length and MTC and discrete virtual targets on a monitor installed in front of a treadmill. Our finding did not support our hypothesis (the accuracy of step length and MTC adaptation would be the same between older adults without diabetes and older adults with diabetes who had not developed diabetes-related neuropathy). Thus, diabetes mellitus impaired step length and MTC adaptation.

Diabetes increased the step length and MTC errors without impacting the tendency of responses. This meant that mean constant error signs were positive in all groups in response to all targets except long step length targets. The older adults with diabetes had increased absolute errors in foot displacement adaptation in the sagittal plane compared with older and younger adults. Like other groups, the older group with diabetes had positive mean constant errors when they walked with shorter steps and higher MTC heights and a negative mean constant error during step lengthening. The signs of mean constant errors in older people with diabetes and participants in other groups were similar.

Differences between groups did not originate in the differences between the magnitudes of targets, because they were subject-specific. Previous research suggested that increased errors were related to augmented physiological noises in the sensory-motor system in adults with diabetes [32], frontal and parietal cortex lesions in stroke patients [33], lateralised right hemisphere [34], and reduced abilities to control the development of force in response to tasks [35]. The frontal cortex executes response inhabitation which is important to avoid falling by stopping ongoing commands and modulating them based on sensory information [36, 37]. However, the underlying mechanisms were studied in people with Parkinson’s disease and adults following a stroke might not apply to older adults with diabetes.

In line with previous research [15], the participants reduced their foot displacement errors in response to predictable targets during the online correction when a target stayed on. Participants made voluntary adjustments to their performance when seeing targeted step length or MTC. They decided and reprogrammed their responses [38]. The forward model enabled the central nervous system to predict the consequences of motor commands by modulating feedback loops. The interaction of diabetes and age impairs the central nervous system responsible for processing and integrating information [39-41]. Impaired neural networks increase the latencies of evoked potentials during walking and reduce the conduction velocity in peripheral nerves [42]. Therefore, from a motor planning perspective, older adults with diabetes may need more time to respond compared to people without diabetes with similar accuracy.

The older participants with diabetes used biofeedback and reduced errors of foot trajectory adaptation; however, their errors were larger in magnitudes than the other groups in this study. In line with previous research involving older adults without and with stroke [10, 28, 43–46], this study found that detailed, meaningful information about goal-task performance in each step improved the accuracy of foot displacement adjustments. Like other groups, the older group with diabetes compared their real-time step length and MTC with presented targets and reduced errors in the steps but compared with other groups their errors were significantly larger. These differences might be the result of a delayed comparison between the expected performance and the performance during the online correction [47, 48].

Corticospinal activities increase when visual information is presented [43]. Visual biofeedback increases the firing of corticospinal neurons in the motor cortex and corticospinal pathways [49, 50]. Higher electroencephalography activities during step shortening compared with step lengthening are evident in the difficulty of walking with shorter steps [51].

Increased errors in adaptability tests in the older group with diabetes compared with the control groups might indicate learning deficits. Learning deficits in diabetic rats were found to be associated with changes in the hippocampus and dependent on diabetes duration and severity [52]. However, the older adults with diabetes reduced their errors by comparing the difference between the actual and desired performances in each step, so it was unlikely that the participants had any established learning impairments.

## Conclusions

Increased errors of foot displacement adjustments in older adults with diabetes indicated that underlying diabetes yields an escalated the risk of falling without causing any fall-related injuries. By participating in gait adaptability training programs, older adults with diabetes may manage to improve the accuracy of foot displacement adjustments. The addition of older adults with diabetes with a history of falls would assist in determining the errors of foot displacement that might lead to falls.

## Data Availability

All data are available from the corresponding author, Dr Suzanne Martin.

## Abbreviations

MTC: Minimum toe clearance
MMSE: Mini-Mental State Examination
MNSI: Michigan neuropathy screening instrument

## Declarations

### Informed constent

All participants provided written informed consent.
